# An Improved ASIFT Image Feature Matching Algorithm Based on POS Information

**DOI:** 10.3390/s22207749

**Published:** 2022-10-12

**Authors:** Junchai Gao, Zhen Sun

**Affiliations:** School of Electronic Information Engineering, Xi’an Technological University, Xi’an 710021, China

**Keywords:** UAV image, feature matching, ASIFT algorithm, BRISK descriptor, RANSAC algorithm

## Abstract

The affine scale-invariant feature transform (ASIFT) algorithm is a feature extraction algorithm with affinity and scale invariance, which is suitable for image feature matching using unmanned aerial vehicles (UAVs). However, there are many problems in the matching process, such as the low efficiency and mismatching. In order to improve the matching efficiency, this algorithm firstly simulates image distortion based on the position and orientation system (POS) information from real-time UAV measurements to reduce the number of simulated images. Then, the scale-invariant feature transform (SIFT) algorithm is used for feature point detection, and the extracted feature points are combined with the binary robust invariant scalable keypoints (BRISK) descriptor to generate the binary feature descriptor, which is matched using the Hamming distance. Finally, in order to improve the matching accuracy of the UAV images, based on the random sample consensus (RANSAC) a false matching eliminated algorithm is proposed. Through four groups of experiments, the proposed algorithm is compared with the SIFT and ASIFT. The results show that the algorithm can optimize the matching effect and improve the matching speed.

## 1. Introduction

Unmanned aerial vehicles (UAV) have been widely used in military and civil fields due to their strong mobility and low cost. However, the 3D modeling of objects constructed via UAV aerial photography is not mature enough in various fields [1]. Therefore, it is of great practical significance to study the 3D modeling of UAV images [2]. The stability of the feature points extracted via image matching and the matching accuracy of the feature point pairs affect the accuracy of 3D reconstruction model to a large extent. Therefore, the feature matching is a key link in the 3D modeling of UAV images [3]. 

In the past two decades, new feature point extraction and description algorithms have been proposed and applied to UAV image matching. Since Lowe proposed the scale-invariant feature transform (SIFT) algorithm [4] in 1999, it has attracted a lot of research and led to a series of feature extraction algorithms. Regarding the sped-up robust features (SURF) algorithm [5] proposed at the ECCY conference in 2006, when compared with the SIFT algorithm, its computing speed is greatly improved and the rotation and image fuzzy invariance robustness is better, but the perspective transformation is not ideal. In 2009, MOREL JM et al. proposed the ASIFT (affine SIFT) algorithm [6]. Compared with the SIFT and its improved algorithm, the ASIFT algorithm has a better matching effect for remote sensing images with scale changes, rotation, and illumination changes. However, the complexity of the ASIFT algorithm is almost twice that of the SIFT algorithm [7], and there are problems with the slow matching efficiency and greater number of mismatching points. In [8], in order to solve the problem of the slow efficiency caused by the sampling of multiple camera axis parameters when the ASIFT algorithm simulates image distortion, an inverse affine transformation is proposed for the original tilt image to recover the approximate orthophoto at one time, which improves the efficiency of the ASIFT algorithm. In [9], in order to solve the problems of the time-consuming process and high false matching degree of the SIFT algorithm, a fast feature matching algorithm based on the fusion of the quad-tree SIFT and K-D tree is proposed to achieve high matching efficiency. However, the high complexity of the 128-dimensional descriptors in the ASIFT is not considered, resulting in low algorithm efficiency. The BRISK (binary robust invariant scalable keypoints) algorithm [10] was proposed by Stefan et al. The efficiency of the algorithm is better than for the SIFT and SURF algorithms. The advantage of the algorithm in terms of the running speed is due to the use of binary feature descriptors. The binary form of the vector can be easily matched using a computer and has faster computing power.

Therefore, aiming to solve the problems of the slow matching efficiency and large number of mismatched point pairs in the ASIFT algorithm [11], the main contents of this paper are divided into the following parts. In the first part, we analyze the problem of the UAV image matching algorithm based on the ASIFT. In the second part, the ASIFT algorithm is improved. In the aspect of the affine image, the known POS information of the UAV is introduced into the simulation of the image distortion to determine the attitude of the image to obtain a simulated image. The idea of traversal is changed to improve the running speed of the algorithm when affine images are used. When describing the features, combined with the BRISK algorithm, the 128-dimensional descriptor is replaced by a binary descriptor, which reduces the memory size and improves the efficiency of the algorithm in feature point matching. In the feature point matching part, the NNDR and RANSAC algorithms are used to eliminate the mismatched point pairs to solve the mismatched feature points. In the third part, we conduct experiments to verify the matching efficiency and matching point pair accuracy of the improved algorithm.

## 2. Problem Analysis of Image Matching Algorithm

### 2.1. Efficiency of ASIFT Algorithm

(1) Analysis of the number of simulated images

The ASIFT algorithm uses an affine transformation to achieve the perspective transformation of the image. By sampling the camera pose when the input image is taken, the simulated images under each perspective are obtained. This series of simulated image sets is used for large-scale matching experiments. Finally, the matching results are statistically compared to obtain the optimal matching. The ASIFT affine transformation principle is as follows:

① Construction of the affine model

The first step of the ASIFT algorithm is to transform the image, using horizontal and vertical angles to simulate all possible affine distortions. The affine transformation matrix A can be decomposed into:

(1) $$A=\lambda \left[\begin{array}{cc} \cos\psi & -\sin\psi \\ \sin\psi & \cos\psi \end{array}\right]\left[\begin{array}{cc} t & 0\\ 0 & 1 \end{array}\right]\cdot \left[\begin{array}{cc} \cos\varphi & -\sin\varphi \\ \sin\varphi & \cos\varphi \end{array}\right]$$

**Figure 1:**
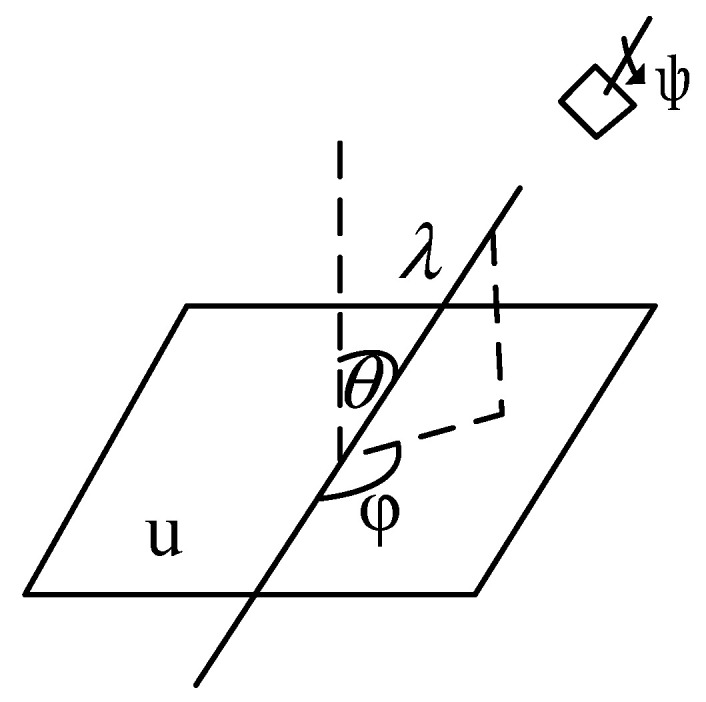
Geometric model of affine camera.

In the formula, λ represents the focal length of the camera, which is the scale magnification, ψ is the rotation angle of the camera, and t and 1 are two eigenvalues of the diagonal matrix of the tilt metric; that is, the first eigenvalue of a diagonal matrix is *t* and the second eigenvalue is 1. Here, φ∈[0,π], φ, and $\theta =\arccos\frac{1}{t}$ are camera viewpoint parameters. The geometric interpretation of the affine camera model is shown in Figure 1:

In Figure 1, the plane where the UAV camera is located is a quadrilateral on the upper right corner, indicating that the camera shoots the object plane at this spatial position; the plane view of the object is represented by u; λ is the scaling factor, φ and θ are the observed angles, and ψ is the rotation parameter of the camera.

② Sampling of latitude and longitude angles

The affine distortion of the image is determined by two variables, the longitude φ and latitude θ. In order to make the ASIFT algorithm invariant to any affine transformation, the tilt t and longitude φ must have higher accuracy. The ASIFT obtains a series of affine simulation images at different camera viewpoints by sampling the camera viewpoint parameters t and φ in the spatial position, where the sampling value $t=1,a,a^{2},\ldots ,a^{n}$, the selected $a=\sqrt{2}$, n=5, and the sampling value longitude angle $\varphi =0,\frac{b}{t},\ldots ,\frac{kb}{t}$, where, b≈72, $\frac{kb}{t}<180^{{}^{\circ}}$, and k are integers.

The ASIFT algorithm can effectively solve the problem of large-angle differences in UAV images, but it takes a lot of memory to simulate affine images of different angles and to match multiple images at the same time. The speed of the algorithm is too slow compared with other matching algorithms. Therefore, although the ASIFT algorithm shows superior performance, the problem of slow efficiency due to multiple sampling is still prominent. Therefore, this paper improves the simulation from the perspective of the ASIFT and proposes a method based on POS data to obtain the attitude information from the UAV camera at the moment of shooting. The attitude information is affined to an analog image to avoid multiple sampling and blind traversal. This approach improves the running speed of the algorithm.

(2) Feature descriptor problem analysis

**Figure 2:**
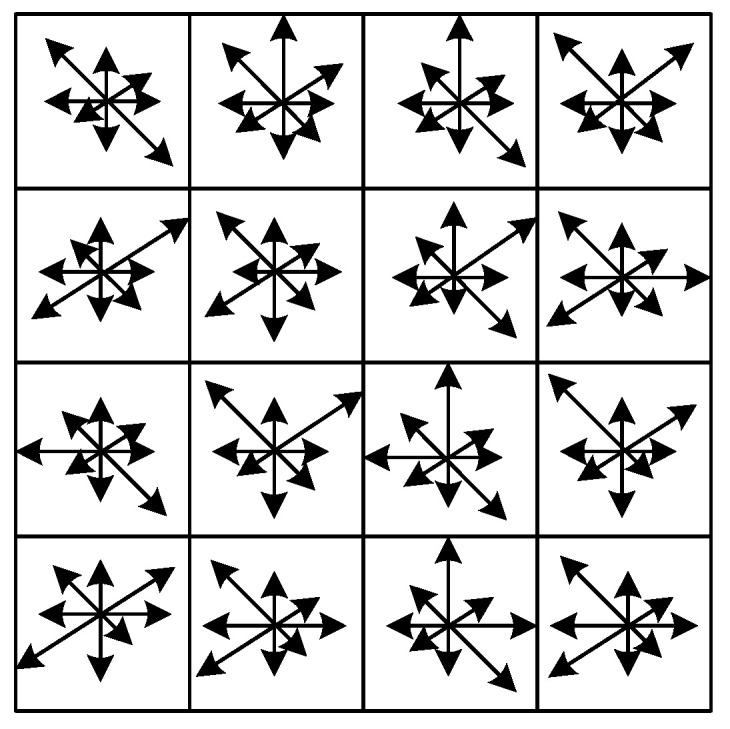
The 128-dimensional descriptor generation graph.

After the image distortion is simulated, the SIFT feature points are extracted and described for the simulated image, and the core of the ASIFT algorithm is the SIFT. The SIFT algorithm uses 128-dimensional vectors as descriptors. The generation diagram of the feature descriptors is shown in Figure 2, which is a high-dimensional feature descriptor. Since SIFT descriptors are composed of floating-point data, 512 bytes are needed, which not only consumes time in the generation of descriptors but also takes up a lot of memory, so that the computational complexity of the feature matching is high [12], resulting in a longer matching time.

Therefore, in order to solve the problem of low efficiency in the process of the generation and matching of constructed descriptors in the algorithm, The BRISK descriptors are represented by a 512-bit binary number [13], which is only 64 bytes and has the advantage of low memory occupancy. Compared with the 512 bytes occupied by the SIFT floating-point descriptors, the BRISK descriptors have greater computing power and are easier to match with computers.

### 2.2. Algorithm Mismatch Analysis

Matching the BRISK binary descriptors involves a simple calculation of the hamming distance of two descriptors via bitwise XOR, whereby the same bit number is their similarity measure. After calculating the hamming distance matching, due to the change in image perspective and the approximate descriptors, there will be more mismatches in the matching results. Moreover, because the feature descriptors used are local feature descriptors, the lack of utilization of the overall information of the image will also lead to mismatches. If these mismatch pairs are not removed, the subsequent image matching will reduce the accuracy due to the deviation of the model transformation. Therefore, it is necessary to purify the mismatched points, so the NNDR algorithm is used to eliminate the coarse matching. After a preliminary screening of the matching points, the mismatch phenomenon cannot be avoided under noise and other interferences. In this paper, the RANSAC algorithm is used to purify the mismatched feature points and to eliminate the mismatches, so as to complete the high-precision image matching [14,15].

## 3. Image Matching Design Based on Improved ASIFT Algorithm

**Figure 3:**
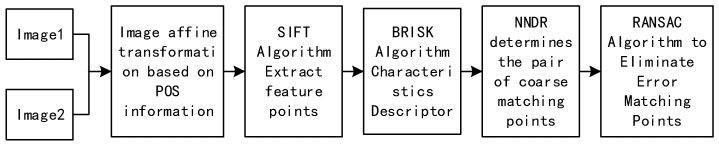
Improved ASITT algorithm flow.

Aiming to solve the problem of the slow matching efficiency and greater number of mismatching points in the ASIFT algorithm, a simulated image is obtained using the known POS information from the UAV to reduce the time consumed for multiple sampling parameters. Then, the advantages of the high robustness of the feature points of the SIFT algorithm in scale space and the high matching efficiency of the BRISK feature descriptor in the Hamming space are used to achieve improvements, so that both the accuracy and efficiency are taken into account. Through the double detection of the NNDR algorithm and RANSAC algorithm, the mismatch is eliminated to improve the matching accuracy. The algorithm flow chart is shown in Figure 3.

### 3.1. ASIFT Image Affine Transformation Based on UAV POS Data

**Figure 4:**
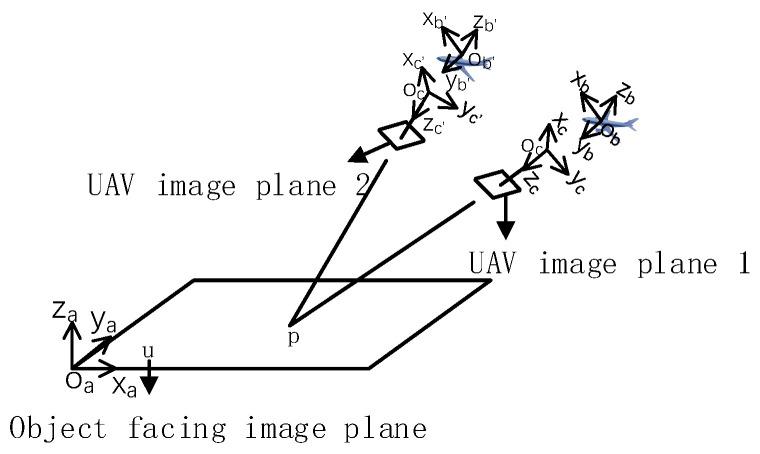
The simple constructed model.

The ASIFT algorithm simulates multiple affine results between the UAV image and the object plane view via multiple sampling of the UAV camera position. Therefore, based on this concept, the UAV POS information is proposed to assist in simulating image distortion. The UAV POS data include the attitude information from the UAV and the UAV camera. When the UAV takes an image, it is recorded in the image information. Therefore, the heading angle, pitch angle, and roll angle information of the UAV and the azimuth angle and pitch angle information from the camera are available. The rotation matrix between the coordinate systems is calculated using the obtained camera and body attitude, and the affine transformation matrix between UAV image 1 and UAV image 2 is solved to obtain an affine image of UAV image 1 simulating the angle of UAV image 2. The ASIFT algorithm changes the process of repeatedly sampling the camera parameters to simulate a series of affine images. The simple model constructed is shown in Figure 4.

Figure 4 shows two drone cameras shooting the object plane in this pose in a spatial position. The reference coordinate system in the figure is the WGS84 coordinate system, which is expressed as $O_{a}-X_{a}Y_{a}Z_{a}$. The coordinates of the two UAV cameras are expressed as $O_{c}-X_{c}Y_{c}Z_{c}$ and $O_{c}-X_{c^{\prime }}Y_{c^{\prime }}Z_{c^{\prime }}$ respectively. The airborne coordinate systems of the two UAVs are expressed as $O_{b}-X_{b}Y_{b}Z_{b}$ and $O_{b^{\prime }}-X_{b^{\prime }}Y_{b^{\prime }}Z_{b^{\prime }}$ respectively.

According to the principle of camera imaging, in Figure 3, from the perspective of UAV camera imaging, the relationship between the two coordinate systems of the object plane and UAV image 1 is analyzed:

(2) $$\begin{array}{l} \left[\begin{array}{c} u_{p}^{a}\\ v_{p}^{a}\\ 1 \end{array}\right]=s^{a}K_{3\times 3}^{a}\left[\begin{array}{cc} R_{3\times 3}^{a} & T_{3\times 3}^{a} \end{array}\right]\left[\begin{array}{c} x_{p}^{g}\\ y_{p}^{g}\\ z_{p}^{g}\\ 1 \end{array}\right]\\ \;{=H}_{3\times 3}^{a}\left[\begin{array}{c} x_{p}^{g}\\ y_{p}^{g}\\ z_{p}^{g}\\ 1 \end{array}\right] \end{array}$$

In the formula, $u_{p}^{a}$ and $v_{p}^{a}$ are the coordinates of point p in the camera coordinate system and point $x_{p}^{g}$, $y_{p}^{g}$, $z_{p}^{g}$ in the reference coordinate system, respectively; $s^{b}$ is the scale factor, $K_{3\times 3}^{a}$ is the inner orientation element of the UAV camera, G and H are the outer orientation elements of the UAV camera, and I is the homography moment of the plane where $H_{3\times 3}^{a}$ is located for UAV image 1. Similarly, the relationship between the two coordinate systems of the p point on the object plane and UAV image 2 is as follows:

(3) $$\begin{array}{l} \left[\begin{array}{c} u_{p}^{b}\\ v_{p}^{b}\\ 1 \end{array}\right]=s^{b}K_{3\times 3}^{b}\left[\begin{array}{cc} R_{3\times 3}^{b} & T_{3\times 3}^{b} \end{array}\right]\left[\begin{array}{c} x_{p}^{g}\\ y_{p}^{g}\\ z_{p}^{g}\\ 1 \end{array}\right]\\ \;{=H}_{3\times 3}^{b}\left[\begin{array}{c} x_{p}^{g}\\ y_{p}^{g}\\ z_{p}^{g}\\ 1 \end{array}\right] \end{array}$$

In Equations (2) and (3), $H_{3\times 3}^{a}$ and $H_{3\times 3}^{b}$ represent the plane where p is located and the homography matrix of the two UAV images, respectively. From the above two equations, we can get:

(4) $$\begin{array}{l} \left[\begin{array}{c} u_{p}^{a}\\ v_{p}^{a}\\ 1 \end{array}\right]=H_{3\times 3}^{a}{\left[H_{3\times 3}^{b}\right]}^{-1}\left[\begin{array}{c} u_{p}^{b}\\ v_{p}^{b}\\ 1 \end{array}\right]\\ \;{=H}_{3\times 3}^{ab}\left[\begin{array}{c} u_{p}^{b}\\ v_{p}^{b}\\ 1 \end{array}\right] \end{array}$$

The plane where point p is located is the intermediate variable for the conversion between two UAV images. The affine homography matrix $H_{3\times 3}^{ab}$ between UAV image 1 and UAV image 2 is obtained from Equations (2) and (3). $H_{3\times 3}^{ab}$ represents the mapping relationship between a point in UAV image 1 and UAV image 2. Therefore, the affine perspective transformation is performed between the two UAV images through $H_{3\times 3}^{ab}$:

(5) $$H_{3\times 3}^{ab}=s^{a}s^{-b}K_{3\times 3}^{a}K_{3\times 3}^{-b}C_{c}^{c^{\prime }}$$

**Figure 5:**
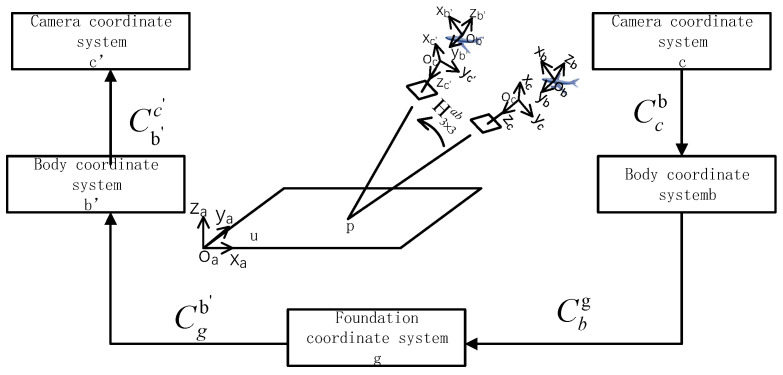
Coordinate transformation diagram.

The matrix shows that it can show the mapping relationship between point p in the two UAV images; $s^{a}$, $s^{b}$, $K_{3\times 3}^{a}$, and $K_{3\times 3}^{-b}$ are obtained using Zhang Zhengyou’s camera calibration experiment. $C_{c}^{c^{\prime }}$ is the conversion relationship between UAV camera coordinate system $O_{c}-X_{c}Y_{c}Z_{c}$ and UAV camera coordinate system $O_{c}-X_{c^{\prime }}Y_{c^{\prime }}Z_{c^{\prime }}$, which is realized by using multiple coordinate conversions. The coordinate transformation diagram is shown in Figure 5.

(6) $$\begin{array}{l} C_{c}^{c^{\prime }}=C_{g}^{c^{\prime }}C_{c}^{g}\\ \;\;=C_{b^{\prime }}^{c^{\prime }}C_{g}^{b^{\prime }}C_{b}^{g}C_{c}^{b} \end{array}$$

In the formula, $C_{c}^{g}$ is the transformation matrix of the UAV camera coordinate system $O_{c}-X_{c}Y_{c}Z_{c}$ to the reference coordinate system and $C_{g}^{c^{\prime }}$ is the transformation matrix of the reference coordinate system to the UAV camera coordinate system $O_{c}-X_{c^{\prime }}Y_{c^{\prime }}Z_{c^{\prime }}$. These can be derived from the coordinate system transformation:

(7) $$C_{g}^{c^{\prime }}={\left[\begin{array}{ccc} 1 & 0 & 0\\ 0 & \cos\beta^{\prime } & \sin\beta^{\prime }\\ 0 & -\sin\beta^{\prime } & \cos\beta^{\prime } \end{array}\right]}^{-1}{\left[\begin{array}{ccc} \cos\alpha^{\prime } & 0 & -\sin\alpha^{\prime }\\ 0 & 1 & 0\\ \sin\alpha^{\prime } & 0 & \cos\alpha^{\prime } \end{array}\right]}^{-1}{\left[\begin{array}{ccc} 1 & 0 & 0\\ 0 & 0 & 1\\ 0 & -1 & 0 \end{array}\right]}^{-1}\left[C_{g}^{b^{\prime }}\right]$$

(8) $$C_{c}^{g}=\left[\begin{array}{ccc} 1 & 0 & 0\\ 0 & \cos\beta & \sin\beta \\ 0 & -\sin\beta & \cos\beta \end{array}\right]\left[\begin{array}{ccc} \cos\alpha & 0 & -\sin\alpha \\ 0 & 1 & 0\\ \sin\alpha & 0 & \cos\alpha \end{array}\right]\left[\begin{array}{ccc} 1 & 0 & 0\\ 0 & 0 & 1\\ 0 & -1 & 0 \end{array}\right]{\left[C_{g}^{b}\right]}^{-1}$$

where α is the azimuth angle of the UAV camera to the body when shooting image 1 and β is the pitch angle; $\alpha^{\prime }$ is the azimuth angle to the body when the drone camera takes image 2 and $\beta^{\prime }$ is the pitch angle. Here, $C_{g}^{b}$ represents the conversion matrix of the reference coordinate system to the UAV body coordinate system $O_{b}-X_{b}Y_{b}Z_{b}$ and $C_{g}^{b^{\prime }}$ represents the conversion matrix of the reference coordinate system to the UAV body coordinate system $O_{b^{\prime }}-X_{b^{\prime }}Y_{b^{\prime }}Z_{b^{\prime }}$. The POS data from of the UAV include the attitude information for the UAV and the UAV camera, which is recorded in the image information when the UAV takes the image. Therefore, the heading angle, pitch angle, and roll angle information for the UAV and the azimuth angle and pitch angle information for the camera are available. The heading angle of the UAV obtained from the POS data of the UAV when shooting image 1 is ψ, the pitch angle is θ, and the roll angle is γ. When shooting image 2, the heading angle is $\psi^{\prime }$, the pitch angle is $\theta^{\prime }$, and the roll angle is $\gamma^{\prime }$.

(9) $$C_{g}^{b}=\left[\begin{array}{ccc} \cos\gamma & 0 & \sin\gamma \\ 0 & 1 & \sin\gamma \\ \sin\gamma & 0 & \sin\gamma \end{array}\right]\left[\begin{array}{ccc} 1 & 0 & 0\\ 0 & \cos\theta & \sin\theta \\ 0 & -\sin\theta & \cos\theta \end{array}\right]\left[\begin{array}{ccc} \cos\psi & \sin\psi & 0\\ -\sin\psi & \cos\psi & 0\\ 0 & 0 & 0 \end{array}\right]$$

(10) $$C_{g}^{b^{\prime }}=\left[\begin{array}{ccc} \cos\gamma^{\prime } & 0 & \sin\gamma^{\prime }\\ 0 & 1 & \sin\gamma^{\prime }\\ \sin\gamma^{\prime } & 0 & \sin\gamma^{\prime } \end{array}\right]\left[\begin{array}{ccc} 1 & 0 & 0\\ 0 & \cos\theta^{\prime } & \sin\theta^{\prime }\\ 0 & -\sin\theta^{\prime } & \cos\theta^{\prime } \end{array}\right]\left[\begin{array}{ccc} \cos\psi^{\prime } & \sin\psi^{\prime } & 0\\ -\sin\psi^{\prime } & \cos\psi^{\prime } & 0\\ 0 & 0 & 0 \end{array}\right]$$

By bringing Equations (6)–(9) into Equation (5), the perspective transformation matrix between UAV image 1 and UAV image 2 can be solved, and two UAV image conversion perspective simulation images can be generated.

### 3.2. Feature Extraction and Description of UAV Image Based on the Improved ASIFT

(1) Feature point extraction

① Scale space generation

The establishment of the image scale space makes the extracted image information richer and the features more prominent, which is the primary task of detecting extreme points. The image scale space L(x, y,σ) can be convolved by a constantly changing Gaussian function G(x, y,σ) and the original image I(x, y), as shown below:

(11) L(x,y,σ)=G(x,y,σ)∗I(x,y)

(12) $$G\left(x,y,\sigma \right)=\frac{1}{2\pi \sigma^{2}}e^{-(x^{2}+y^{2})/2\sigma^{2}}$$

In the formula, (x,y) is the coordinates of a point in the image, σ is the scale space factor, and the size is proportional to the scale. In order to detect stable feature points, the convolution calculation between Gauss difference operators and images of different scales is carried out to obtain the extremum points of the Gauss difference scale space. The calculation formula is:

(13) D(x,y,σ)=L(x,y,kσ)−L(x,y,σ)

In the formula, D(x,y,σ) is the extreme point of the scale space of the Gauss difference, L(x,y,σ) is the scale space of the image, and k is the ratio of the adjacent scale factors. On the Gaussian difference pyramid, each pixel in the image is compared with 26 points in the neighborhood of the upper, lower, and same scale layers to ensure that the extreme points supporting the scale invariance can be detected.

② Location of feature points

After obtaining all of the roughly selected extreme point sets, in order to obtain more accurate feature points, it is necessary to use the Taylor series expansion of the Gaussian difference pyramid function in the scale space for the interpolation search. At the same time, the feature points with low contrast are removed as follows:

(14) $$D\left(X\right)=D+\frac{\partial D^{T}}{\partial X}X+\frac{1}{2}X^{T}\frac{\partial^{2}D}{\partial X^{2}}X$$

Among them, $X={\left(x,y,\sigma \right)}^{T}$. Because the process of extracting feature points will produce an edge response, according to the characteristics of edge response points with large principal curvature ratios, the edge response points can be filtered by the threshold of the principal curvature ratio.

③ Determination of the direction of feature points

Taking the feature points as the center, the gradient direction of the feature points is counted by the gradient histogram in the neighborhood. The direction corresponding to the peak of the gradient histogram is the main direction of the feature points.

(2) UAV image feature description based on BRISK

After extracting the feature points in the image, the feature points need to be described to facilitate subsequent matching. The BRISK descriptor description method uses the neighborhood sampling mode; that is, multiple concentric circles are constructed in the 40 × 40 pixel block centered on the feature, and the sampling points are equidistantly distributed on the circle, giving a total of 60. In addition, in order to eliminate the influence of the aliasing effect, Gaussian smoothing filtering is needed for each point.

Let Ω be the sampling point pair set, while the Euclidean distance is used to define the short-distance sampling point pair set S and the long-distance sampling point pair set Q:

(15) $$S=\{(p_{i},p_{j})\in \Omega |p_{j}-p_{i}<\delta_{max}\}\subseteq \Omega$$

(16) $$Q=\{(p_{i},p_{j})\in \Omega |p_{j}-p_{i}>\delta_{min}\}\subseteq \Omega$$

Here, $p_{i}$ and $p_{j}$ are 1 pair of sampling points; two distance thresholds $\delta_{max}=9.75\;t$ and $\delta_{min}=13.67\;t$ are divided into a short-distance S set and long-distance Q set, and t is the scale of the feature point. Let N denote the number of elements in the set Q of the long-distance sampling point pair and $g\left(p_{i},\;p_{j}\right)$ denote the gradient of the point pair $\left(p_{i},\;p_{j}\right)$.

(17) $$g=\left(\begin{array}{c} g_{x}\\ g_{y} \end{array}\right)=\frac{1}{N}\sum_{(p_{i},p_{j})\in P}g\left(p_{i},p_{j}\right)$$

In the formula, $g_{x}$ and $g_{y}$ are the gradient values of the sampling point pairs in the *x* and *y* directions. *P* is the set of long-distance sampling points. *N* is the point-to-set number. When sampling, the rotation invariance can be obtained by rotating the angle around the feature point. The rotated S-set pair is expressed as $\left(p_{i}^{\alpha },p_{j}^{\alpha }\right)$, and finally a 512-bit binary code is formed. The definition is as follows:

(18) $$\begin{array}{l} b=\{\begin{array}{c} 1,I(p_{j}^{\alpha },\sigma_{j})>I(p_{i}^{\alpha },\sigma_{i})\\ 0,\;\mathrm{otherwise} \end{array}\\ \forall (p_{i}^{\alpha },p_{j}^{\alpha })\in S \end{array}$$

In the formula, $p_{j}^{a}$ and $p_{i}^{a}$ are new sampling points obtained by rotating the α angle; $\sigma_{i}$ and $\sigma_{j}$ are the standard deviations of the Gaussian function when the sampling point pair positions i and j.

### 3.3. Feature Point Matching and Purification

(1) Screening of UAV image coarse matching based on NNDR

Feature matching is the process of measuring the similarity of the generated BRISK descriptors and judging the matching relationship according to the preset threshold. At present, the common image matching strategies include the fixed threshold criterion, left–right consistency criterion (LRC), and NNDR criterion. The fixed threshold criterion artificially sets a fixed threshold. Since the feature moves to different positions of the feature space, the effective range of the threshold will change greatly, so the matching result obtained by the fixed threshold will have a high mismatching rate. Compared with other common matching criteria, the LRC can obtain a higher correct matching rate and lower error rate. However, when there are many similar textures in the image, this leads to matching errors and generates mismatches. NNDR is applied in most point matching algorithms. The larger the NNDR value, the more feature matching pairs are obtained but the greater the possibility of mismatching; that is, the matching accuracy will be reduced. Therefore, it is particularly important to select different NNDR values. Combining the advantages and disadvantages of the three measurements and a large number of experiments, in view of the uniqueness of the NNDR feature description, this paper selects NNDR to filter the matching set for the first time. The specific process of the NNDR algorithm is:

① Calculate the Hamming distance

Two BRISK descriptors are matched using the Hamming distance matching technique. The distance D of two feature points is obtained via bit XOR summation and the similarity between two images is calculated:

(19) $$D=(a,b)=\sum_{i=1}^{512}m_{i}⊕n_{i}$$

where D represents the distance between two feature points a and b, $m_{i}$ is the binary number of feature point a, and $n_{i}$ is the binary number of feature point b.

② Distance ratio setting

The UAV image similarity detection technology based on NNDR uses the K-d tree to search the nearest neighbor and second-nearest neighbor for all feature points. According to the ratio of the hamming distance of the nearest neighbor point descriptor to the hamming distance of the next nearest neighbor point descriptor, a screening threshold is set. When the hamming distance of the two feature point descriptors is less than this threshold, it is regarded as the best matching point pair to further improve the image similarity detection.

(20) $$\frac{D_{1}}{D_{2}}\leq D_{threshold}$$

In the formula, $D_{1}$ refers to the nearest neighbor Hamming distance of a feature point in the image to be registered and the nearest neighbor Hamming distance of a feature point in the reference image, $D_{2}$ is the second-nearest neighbor Hamming distance of the feature point, and $D_{threshold}$ is the set screening threshold.

The NNDR algorithm sets a threshold to perform rough matching on the matching points. After the initial matching of the nearest neighbor and the second-nearest neighbor Hamming distance, the matching point pair set is obtained.

(2) Purification of UAV Image Matching Points Based on RANSAC

Although the matching effect is improved after screening the matching point pairs, there are still some mismatches due to noise and other interference, which need to be further purified by the corresponding algorithms. In this paper, the RANSAC algorithm is used to eliminate mismatched point pairs and to improve the matching accuracy. The principle idea is to calculate the mathematical model parameters of the data according to a set of sample data sets containing abnormal data, so as to obtain effective sample data.

The optimal homography 3×3 matrix *H* is introduced to maximize the matching feature points satisfying the matrix. Usually, *h*_9_ = 1 so as to normalize the matrix. The purpose of the algorithm is to find the optimal matrix so that the number of data points satisfying the matrix is maximal.

(21) $$H=\left(\begin{array}{ccc} h_{1} & h_{2} & h_{3}\\ h_{4} & h_{5} & h_{6}\\ h_{7} & h_{8} & h_{9} \end{array}\right)$$

The RANSAC algorithm randomly selects four samples from the matching dataset and ensures that the four samples are not collinear. Through calculation and continuous iterations, the optimal parameter model is found. In this optimal model, the feature points can be matched.

(22) $$\sum_{i=0}^{n}(x_{i}^{\prime }-\frac{h_{11}x_{i}+h_{12}y_{i}+h_{13}}{h_{31}x_{i}+h_{32}y_{i}+h_{33}}{)}^{2}+(y_{i}^{\prime }-\frac{h_{21}x_{i}+h_{22}y_{i}+h_{23}}{h_{31}x_{i}+h_{32}y_{i}+h_{33}}{)}^{2}$$

After the optimal model is obtained, the feature points that do not conform to the optimal model are called ‘outside points’, which will be eliminated.

After matching the feature points, the NNDR algorithm is used to filter the rough matching, and the RANSAC algorithm is used to purify the mismatched points and to eliminate the mismatch.

## 4. Experimental Analysis

### 4.1. Experimental Images

**Figure 6:**
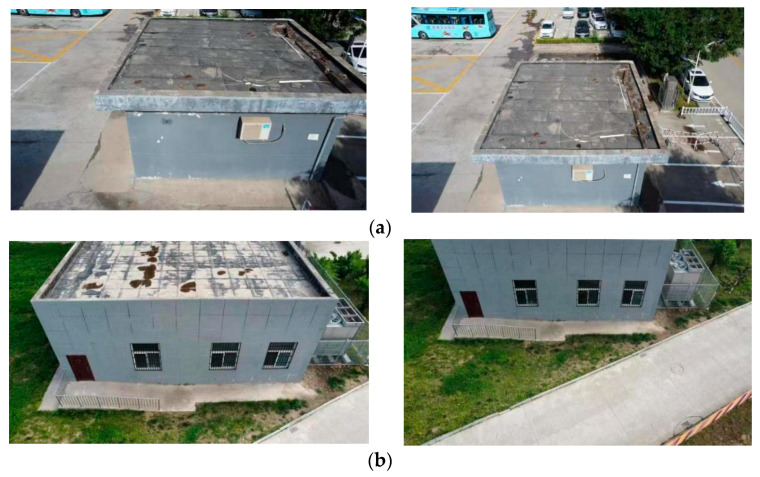
Scale change images. (**a**) Experiment 1 image. (**b**) Experiment 2 image.

**Figure 7:**
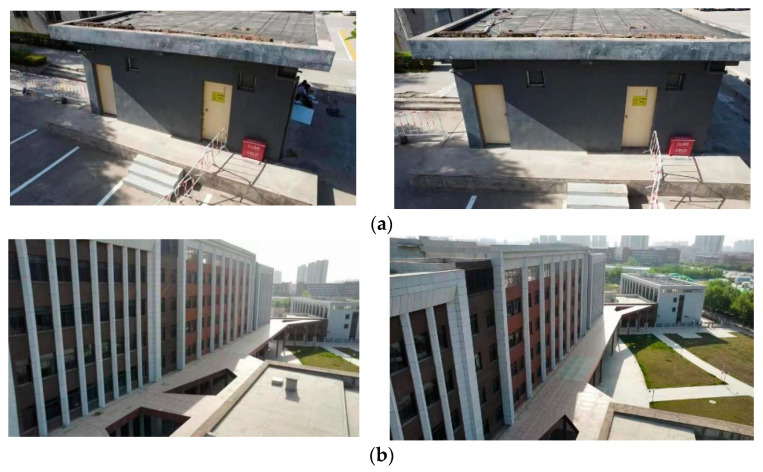
Rotation change images. (**a**) Experiment 3 image. (**b**) Experiment 4 image.

In order to verify the effectiveness of the algorithm, a DJI UAV is used to collect images around and above buildings on campus, and two groups of images with scale and rotation changes are obtained for the experiments, such as in Figure 6 and Figure 7. The data are used for the experiments to verify the matching results of the algorithm for scale and rotation change images using Visual Studio2017 with Windows 10 operating system, with the OpenCV4.1.0 library. The specific step in the algorithm is to use the Pos information from the UAV to simulate image distortion, then to use SIFT to detect the feature points and the BRISK descriptor to describe the extracted feature points to generate the feature descriptor. The rough matching is filtered according to NNDR, and false matching points are eliminated based on the RABSAC algorithm.

### 4.2. Algorithm Matching Results and Analysis

**Figure 8:**
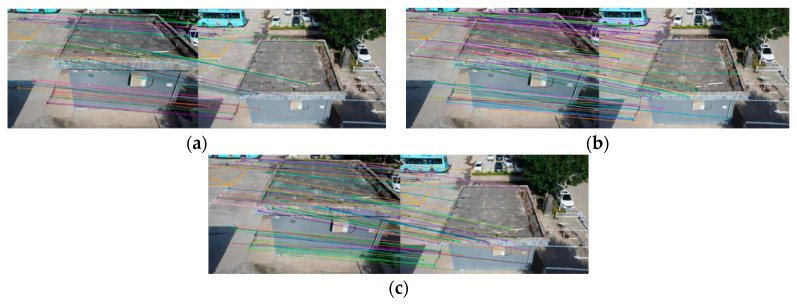
Matching results from experiment 1. (**a**) SIFT algorithm. (**b**) ASIFT algorithm. (**c**) Improved ASIFT algorithm.

**Figure 9:**
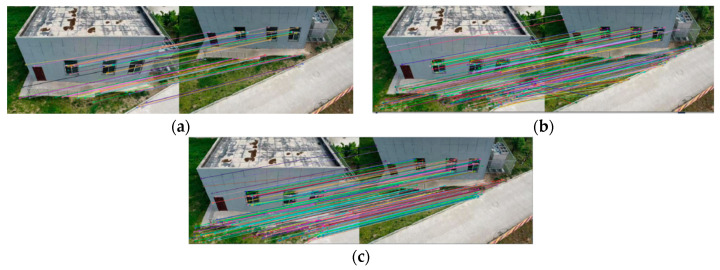
Matching results from experiment 2. (**a**) SIFT algorithm. (**b**) ASIFT algorithm. (**c**) Improved ASIFT algorithm.

**Figure 10:**
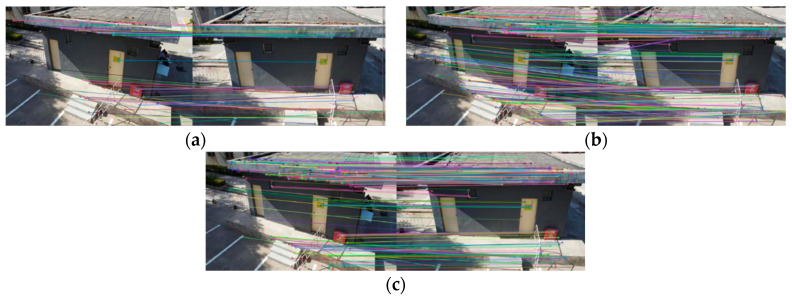
Matching results from experiment 3. (**a**) SIFT algorithm. (**b**) ASIFT algorithm. (**c**) Improved ASIFT algorithm.

**Figure 11:**
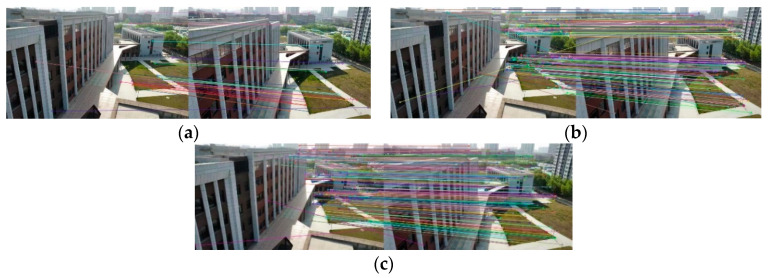
Matching results from experiment 4. (**a**) SIFT algorithm. (**b**) ASIFT algorithm. (**c**) Improved ASIFT algorithm.

Figure 8, Figure 9, Figure 10 and Figure 11 show the matching result graphs for the SIFT, ASIFT, and our algorithm from top to bottom. The colored dots in the graph are the detected feature points and the matching points connected by the colored lines.

Figure 6, Figure 7, Figure 8 and Figure 9, in the same experimental image matching, it can be seen from the density of the connection line that the matching logarithm obtained by the ASIFT algorithm is greater than that of the SIFT algorithm, and the matching effect is better than that of the SIFT algorithm. It shows that the simulation image distortion in the ASIFT algorithm has an obvious effect on the improvement of the number of UAV image matching points. However, it can be seen from the (b) diagram of the experimental results that the matching points of the ASIFT algorithm are chaotic and there are many mismatching points. Therefore, we improve the ASIFT algorithm by adding the RANSAC algorithm to the ASIFT algorithm. From the (c) experimental results, it can be seen that after adding the algorithm, more mismatching points are eliminated, the chaotic matching results are almost invisible, and more accurate matching results are obtained.

**Figure 12:**
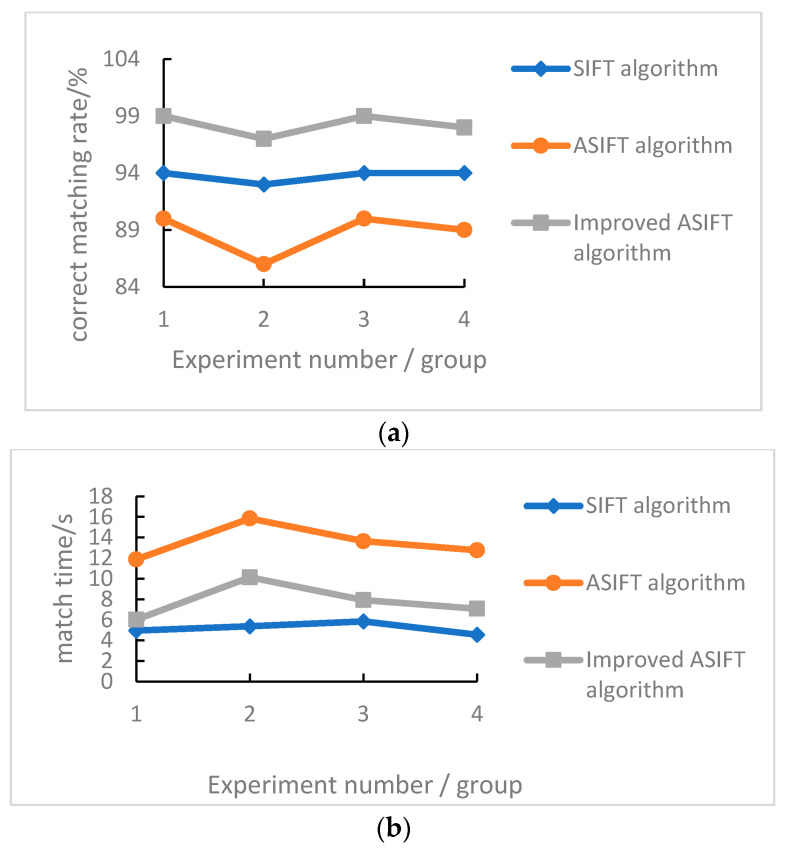
Performance comparison of the three algorithms. (**a**) Comparison of matching accuracy. (**b**) Matching time comparison.

The comparison results for the matching logarithm and matching time of the three algorithms are shown in Figure 12.

It can be seen from Figure 10a that after the error matching is eliminated by the improved ASIFT algorithm, the correct matching rate is much better than that of the ASIFT and SIFT algorithms, reaching more than 95%, while the matching accuracy of the ASIFT algorithm is the lowest. This shows that after the error elimination is added, the error matching is effectively eliminated and the accuracy of the matching point pair is improved. Figure 10b shows that the improved ASIFT algorithm’s matching time is longer than for the SIFT matching algorithm and shorter than for the ASIFT matching algorithm. This shows that the UAV Pos data-aided image and binary descriptor can effectively improve the efficiency of the algorithm matching. Therefore, compared with the ASIFT feature matching algorithm, the improved ASIFT algorithm has a shorter matching time, more correct matching points, and better accuracy.

## 5. Conclusions

The algorithm proposed in this paper is an improved algorithm based on the ASIFT algorithm. The algorithm determines the camera parameters when the simulated image is distorted based on the POS information of the UAV and obtains a frontal simulated image of the UAV image to be matched, then combines the SIFT algorithm. The advantages of the high robustness of the feature points in the scale space and the high matching efficiency of the BRISK feature descriptors in the Hamming space are used for feature extraction and description, so as to maintain the accuracy and effectively improve the time efficiency of the algorithm. Finally, the double mismatch elimination detection of the NNDR algorithm and RANSAC algorithm is carried out so as to improve the matching accuracy. By manipulating the UAV to collect images of four experimental buildings, the matching results of the three algorithms are compared. The experimental results show that when the algorithm of this paper matches the UAV image, the matching point pair has higher accuracy and time efficiency.

## Data Availability

No applicable.
